# Whole-Exome Sequencing and Homozygosity Analysis Implicate Depolarization-Regulated Neuronal Genes in Autism

**DOI:** 10.1371/journal.pgen.1002635

**Published:** 2012-04-12

**Authors:** Maria H. Chahrour, Timothy W. Yu, Elaine T. Lim, Bulent Ataman, Michael E. Coulter, R. Sean Hill, Christine R. Stevens, Christian R. Schubert, ARRA Autism Sequencing Collaboration, Michael E. Greenberg, Stacey B. Gabriel, Christopher A. Walsh

**Affiliations:** 1 Division of Genetics, Department of Medicine, Children's Hospital Boston, Boston, Massachusetts, United States of America; 2 Manton Center for Orphan Disease Research, Children's Hospital Boston, Boston, Massachusetts, United States of America; 3 Howard Hughes Medical Institute, Children's Hospital Boston, Boston, Massachusetts, United States of America; 4 Department of Pediatrics, Harvard Medical School, Boston, Massachusetts, United States of America; 5 Program in Medical and Population Genetics, Broad Institute of Massachusetts Institute of Technology and Harvard University, Cambridge, Massachusetts, United States of America; 6 Department of Neurology, Harvard Medical School, Boston, Massachusetts, United States of America; 7 Biological and Biomedical Sciences Program, Harvard University, Boston, Massachusetts, United States of America; 8 Analytic and Translational Genetics Unit, Massachusetts General Hospital, Boston, Massachusetts, United States of America; 9 Department of Neurobiology, Harvard Medical School, Boston, Massachusetts, United States of America; University of California Los Angeles, United States of America

## Abstract

Although autism has a clear genetic component, the high genetic heterogeneity of the disorder has been a challenge for the identification of causative genes. We used homozygosity analysis to identify probands from nonconsanguineous families that showed evidence of distant shared ancestry, suggesting potentially recessive mutations. Whole-exome sequencing of 16 probands revealed validated homozygous, potentially pathogenic recessive mutations that segregated perfectly with disease in 4/16 families. The candidate genes (*UBE3B*, *CLTCL1*, *NCKAP5L*, *ZNF18*) encode proteins involved in proteolysis, GTPase-mediated signaling, cytoskeletal organization, and other pathways. Furthermore, neuronal depolarization regulated the transcription of these genes, suggesting potential activity-dependent roles in neurons. We present a multidimensional strategy for filtering whole-exome sequence data to find candidate recessive mutations in autism, which may have broader applicability to other complex, heterogeneous disorders.

## Author Summary

Autism spectrum disorders are neurodevelopmental disorders that are genetically highly heterogeneous, with no single gene accounting for more than 1% of cases. In order to identify recessive mutations, we selected probands from an outbred population based on abundance of homozygosity in their genomes. We interrogated the entire coding sequences of 16 probands that had evidence of parental shared ancestry and identified four candidate autism genes. Furthermore, the expression of these genes was responsive to neuronal activity. We present a strategy for identifying candidate recessive mutations in genetically complex disorders.

## Introduction

Autism is a neurodevelopmental disorder characterized by impaired communication skills, social behavior abnormalities, and stereotypies, with a prevalence of ∼1/150 children [1]. It is considered to be one of the most highly genetic neuropsychiatric disorders with a heritability of 40–80% [2], [3]. Family studies show that siblings of autistic children are at a ∼25-fold higher risk to develop autism than the general population [4], and twin studies show concordance of the autism phenotype in 20–30% of dizygotic twins and ∼60% of monozygotic twins [3], [4]. Genome-wide linkage and association studies, and candidate gene approaches have identified several susceptibility loci and implicated potential autism genes [5]–[7]. The fact that no single genetic aberration accounts for more than 1% of cases suggests extreme genetic heterogeneity [8], [9], posing a major challenge to identifying causative genes. To date genes have been identified on the basis of overlap with other syndromic neurodevelopmental disorders (e.g. Fragile X syndrome, Angelman syndrome, Rett syndrome), chromosomal abnormalities and copy number variation, and as causes for nonsyndromic autism (e.g. *NRXN1*, *NLGN3/4X*, *SHANK3*) [4], [10]. In a few cases, autism has been shown to be caused by homozygous recessive mutations due to recent shared ancestry [11], although the contribution of recessive mutations in outbred populations remains unexplored.

Recessive mutations in autism may behave like other rare recessive traits, thus allowing gene mapping using homozygosity analysis. Homozygosity mapping is frequently employed to isolate disease genes in families where the parents are known to be definably related, typically as cousins, which increases the risk for recessive disease [12]–[14]. However homozygous recessive “founder” mutations are also common in patients whose parents share only distant ancestry, common ethnicity, or in some cases no apparent ancestry at all [15], and population analysis of runs of homozygosity has been used to define genomic loci that may harbor such mutations in diseases characterized by genetic heterogeneity [16]–[18]. Here we surveyed the mutational spectrum in individuals with autism from nonconsanguineous populations who were selected for the high degree of homozygosity in the genome, since high levels of homozygosity suggest distant or cryptic shared ancestry of the parents. We identified several patients with potentially new autism mutations, and found that a surprising number of these mutations occurred in genes that are regulated by neuronal depolarization.

## Results/Discussion

**Figure 1:**
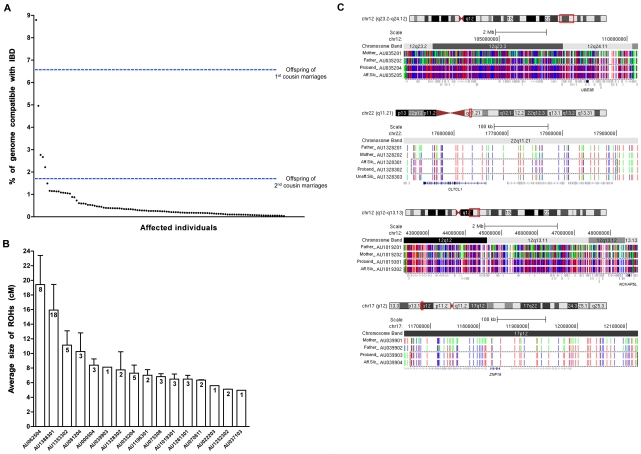
Homozygosity analysis in the AGRE collection. (A) A plot of the percent homozygosity in the genome of probands from the entire AGRE collection. All affected individuals with runs of homozygosity (ROHs) >5 cM are plotted. Offspring of first cousin marriages are expected to have 6.25% homozygosity in their genomes, while those of second cousin marriages are expected to have 1.6%. IBD: identity by descent. (B) The average sizes of the ROHs in cM are plotted for each of the 16 AGRE samples that were sequenced. The number of the ROHs is shown in each bar. Values are mean ± SEM. (C) ROHs containing candidate disease variants are shared by affected individuals and absent from unaffected individuals. Sample names are indicated on the left (Aff.Sib: affected sibling, Unaff.Sib: unaffected sibling). Homozygous SNPs are shown in red or blue and heterozygous SNPs are shown in green. ROHs are enclosed in the dotted box. The candidate autism gene in each family is shown in navy below the ROHs. All other genes in grey did not contain rare, potentially pathogenic variants. No whole genome SNP data is available for individual AU035203, but we genotyped the sample for all homozygous variants identified by the whole exome sequencing of AU035204.

To sort the genetic heterogeneity of autism, we used homozygosity analysis [19] to identify a subset of patients likely to be enriched for recessive mutations. We performed a homozygosity-based analysis of 1000 families (5,431 individuals) in the Autism Genetic Research Exchange (AGRE) [20] cohort. Though most American families in this cohort are of mixed European ancestry and share no acknowledged near ancestors, we hypothesized that a small proportion of European-American parents share a traceable common ancestor, or may share common ethnic ancestry through both parental lines, which in either case may result in homozygosity for rare recessive founder mutations, as has been demonstrated for a host of known Mendelian recessive diseases [21]. We identified a small subset of “outlier” AGRE families (<2% of the total) in which the affected children show runs of homozygosity totaling up to ∼9% of their genome. This low proportion of families with elevated homozygosity is consistent with low reported rates of consanguinity in the AGRE collection. Nonetheless, in the few outlier families, rates of homozygosity are far higher than generally observed in individuals whose parents have no common ancestry (≤1.6%), and overlap or exceed in some cases the predicted range of homozygosity expected in offspring of first cousin parents (6.25%) [22] (Figure 1A). The sizes of homozygous blocks in probands from these outlier families ranged from ∼5–19 cM on average (Figure 1B), suggesting ancient shared ancestry in these families compared to larger blocks of homozygosity seen in consanguineous families (≥20 cM) [22]. Since the AGRE dataset provides no specific information about shared ancestry or consanguinity between parents, we explored the level of shared ancestry between parents, by performing tests to estimate relatedness between individuals based on identical-by-state (IBS) and identical-by-descent (IBD) genotype information [23], [24]. We find that for 16 families where probands had the largest amount of homozygosity in their genomes, some of the parental pairs were more closely related than average (Figure S1), but that parental relatedness by itself, as analyzed by these methods, did not always predict the degree of homozygosity in the offspring.

We performed whole exome sequencing in 16 AGRE patients, selected because they showed the largest total proportion of their genome homozygous (∼1%–9%) of all patients in the collection. We reasoned that some of the runs of homozygosity would contain homozygous causative mutations. Whole exome sequencing allows for the high-throughput, unbiased survey of all exonic variation in a patient, including any known mutations. Sequencing was performed using the Illumina Genome Analyzer II platform following enrichment of exonic sequences using Agilent's SureSelect Human Exome Kit. We obtained an average coverage of 92% at 20X (Table S1), and identified an average of 34,615 total variants per exome (Table S2), subsequently filtering them to identify rare, likely deleterious changes. Since we wanted to identify rare private mutations, common variants identified by the 1000 Genomes project and dbSNP130 were filtered out, and remaining variants were subject to an in-house bioinformatics pipeline to annotate variants that may disrupt gene function (by altering the coding sequence, the splice sites, or truncating the protein). On average, 735 variants per exome were potentially pathogenic, and out of these, 39 per genome (on average) were homozygous (Table S2). The availability of whole exome sequence allowed us to test each patient systematically for mutations in known autism genes on the autosomes as well as the X chromosome, and no inherited mutations that were predicted to be damaging in well-documented autism genes were found in the 16 patients.

**Figure 2:**
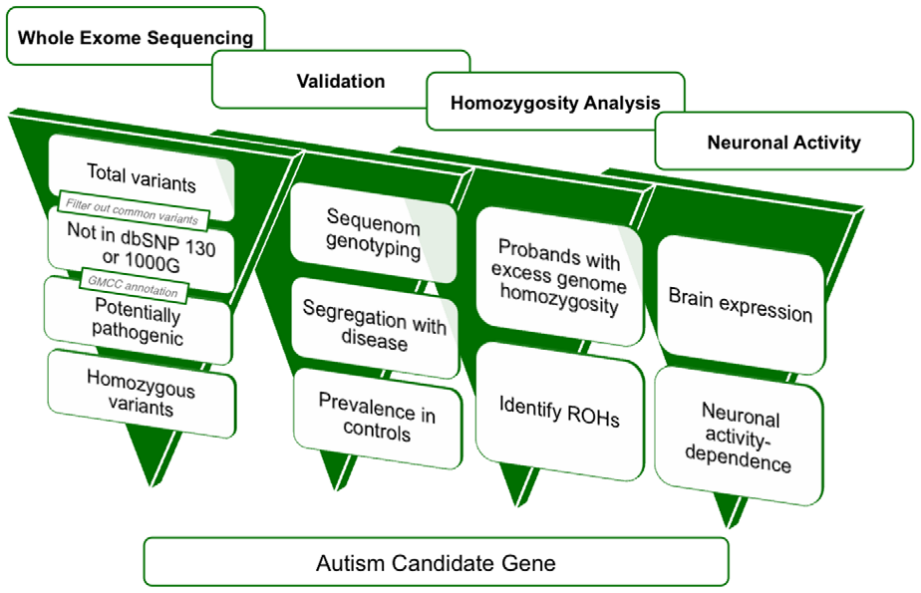
A four-dimensional approach to identifying autism candidate genes. Overview of variant filtration and prioritization of whole exome sequencing data. Results from variant validation and homozygosity analysis were combined with neuronal activity data to identify candidate autism genes from whole exome sequence. 1000G: 1000 Genomes Project, GMCC: genomic mutation consequence calculator, ROHs: runs of homozygosity.

To rule out variants that arose from spontaneous cell line artifacts, somatic mosaic mutations, or sequencing errors, we validated all homozygous variants in all family members using Sequenom technology. Genotyping candidate variants in the 16 probands allowed us to examine inheritance of variants as well as segregation with disease, since many families had multiple affected individuals as well as unaffected siblings (Figure S2). Variants that did not validate with Sequenom genotyping despite high sequencing depth (≥100) generally occurred in regions of the genome that were not uniquely mappable. For uniquely mapped variants, the rate of validation correlated well with sequencing depth (Pearson's correlation = 0.532, *P* = 0.001×10^−30^, *t*-test) (Figure S3). Analysis of segregation further permitted us to focus on bona fide inherited mutations as we only considered those variants that were homozygous in the proband (by whole exome sequencing and Sequenom confirmation), heterozygous or absent in unaffected siblings, and transmitted from heterozygous parents. This validation step thus eliminates any possible sequencing errors or somatic mutations that complicate many high-throughput sequencing studies. We overlaid the validated variants with the result of our homozygosity analysis and further focused our attention on that subset of variants that fell within runs of homozygosity shared by affected siblings and absent from unaffected siblings. This allowed us to narrow down the number of candidate variants per exome, and for four families only 1 variant segregated with the disease (Table 1, Figure 1C). For some families our approach did not yield any candidate recessive variants as expected, since homozygous variants will not necessarily be causative even in some families selected based upon homozygosity. We then further examined the prevalence of candidate homozygous mutations in a control population of ∼700 normal individuals. We were able to exclude homozygous variants based on several criteria including: prevalence in controls, the genes not being expressed in brain, or the genes being mutated in other disorders (Table S3). Under this variant prioritization model (Figure 2), candidate autism mutations were identified in four of the 16 probands (Table 2, Figure 1C), with these candidate disease variants falling within runs of homozygosity shared by affected siblings and absent from unaffected siblings.

**Table 1:** Whole-exome sequencing identifies rare, previously unreported homozygous variants in 16 AGRE autism patients.

Homozygous variants				Validated		
Patient	WES	Sequenom design	Sequenom run	Validated	Segregate with disease	…and in ROHs
AU070811	32	27 (84%)	22 (81%)	10 (45%)	2	1
AU035204	48	40 (83%)	34 (85%)	12 (35%)	5	4
AU081204	29	25 (86%)	20 (80%)	8 (40%)	0	0
AU075308	36	30 (83%)	23 (77%)	11 (48%)	2	1
AU1328302	38	34 (89%)	30 (88%)	15 (50%)	1	1
AU1261301	35	30 (86%)	26 (87%)	15 (58%)	0	0
AU1353302	26	23 (88%)	17 (74%)	9 (53%)	1	0
AU1252302	32	25 (78%)	21 (84%)	9 (43%)	1	0
AU037103	25	24 (96%)	18 (75%)	10 (56%)	1	1
AU1019301	53	45 (85%)	38 (84%)	15 (39%)	3	2
AU1388301	45	40 (89%)	31 (77%)	22 (71%)	0	0
AU1196301	54	49 (91%)	41 (84%)	17 (41%)	0	0
AU022203	40	37 (92%)	34 (92%)	8 (23%)	1	0
AU000504	41	29 (71%)	25 (86%)	13 (52%)	0	0
AU039903	40	33 (82%)	31 (94%)	16 (52%)	2	2
AU062504	43	36 (84%)	28 (78%)	12 (43%)	0	0

ROHs: runs of homozygosity; WES: whole exome sequence.

**Table 2:** Candidate autism genes identified in 4 AGRE patients.

Patient	Gene symbol	Gene name	Mutation	Effect	Prevalence in control chromosomes	PolyPhen-2 prediction (score)	SIFT	Conservation	ROH size (cM)
AU035204	*UBE3B*	Ubiquitin protein ligase E3B	chr12: 108,452,214 C>T	R40C	1/1344 (0.07%) (0 homozygotes)	Probably damaging (1.000)	0	0.75	11.7
AU1328302	*CLTCL1*	Clathrin, heavy chain-like 1	chr22: 17,575,771 G>A	R125C	1/1328 (0.07%) (0 homozygotes)	Probably damaging (0.999)	0	0.72	0.9
AU1019301	*NCKAP5L*	NCK-associated protein 5-like	chr12: 48,476,657 C>T	G11D	0/1340 (0.0%)	Benign (0.004)	0.03	0.61	5.1
AU039903	*ZNF18*	Zinc finger protein 18	chr17: 11,822,517 G>T	H377N	1/1340 (0.07%) (0 homozygotes)	Possibly damaging (0.590)	0.4	0.54	1.1

The Table summarizes genes identified by combined homozygosity mapping and whole exome sequencing, as described in the text. All mutations were homozygous in affected individuals and present within runs of homozygosity (ROH) ranging from 0.9–11.7 cM. All mutations were heterozygous in the parents, while unaffected siblings were either heterozygous or homozygous for the alternate allele. All candidate genes are expressed in the brain. Conservation scores were derived from the UCSC Genome Browser Vertebrate Multiz Alignment and Conservation (17 Species) track.

The candidate mutations identified in this study implicate several candidate genes in autism that encode proteins involved in small GTPase mediated signal transduction, transcriptional regulation, and protein modification processes (Table 2). Among the mutations we identified is a homozygous c.144 C>T change that creates an R40C mutation in ubiquitin protein ligase E3B (UBE3B), a member of the E3 ubiquitin-conjugating enzyme family. UBE3B is highly expressed in the brain and may play a role in stress response [25]. The UBE3B R40C mutation identified in AU035204 is predicted to be damaging, was homozygous in both affected children (monozygotic twins), heterozygous in the parents and unaffected sibling (Figure S2), and was absent in the homozygous state in 1344 control chromosomes. *UBE3B* is highly conserved across species and belongs to the same family as *UBE3A*, the gene disrupted in Angelman syndrome, a neurodevelopmental disorder characterized by intellectual disability, movement or balance problems, abnormal behaviors, and speech and language impairment. Recent work has shown that experience-driven neuronal activity induces *Ube3a* transcription and that it regulates excitatory synapse development and function through targeting the key synaptic molecules Arc and Ephexin5 [26], [27].

We also narrowed down the candidate genes to only one in AU1328302. An R125C mutation in *CLTCL1*, encoding clathrin heavy chain-like 1, was homozygous in both affected children, heterozygous in the parents and unaffected sibling, and predicted to be damaging (Table 2 and Figure S2). *CLTCL1* is disrupted in a patient with features of DiGeorge syndrome, including intellectual disability, facial dysmorphia, long slender digits, and genital anomalies [28]. It encodes a member of the clathrin heavy chain family, representing a major structural component of coated pits and vesicles involved in intracellular trafficking, which are important to glutamate receptor turnover.

Since resequencing of candidate genes in a larger cohort is an important validation step in evaluation of any candidate gene, we screened a larger independent cohort of whole exome data from 418 autism cases and 371 controls, sequenced as part of the ARRA Autism Sequencing Consortium. DNA from these cases and controls underwent whole exome capture, cloning and sequencing in the same fashion that our 16 cases did at the Broad Institute. For all four genes, we compared the rate of mutations under a recessive model, looking for either homozygous or compound heterozygous mutations in cases versus controls. As a group, the 4 genes showed a higher number of recessive mutations (homozygous or compound heterozygous) in cases (24/418, 5.7%) compared to controls (11/371, 3.0%) (*P* = 0.042, Fisher's exact test, one-tailed). These mutations were all missense changes and were relatively rare, all with allele frequencies of ≤5% (Table 3). One gene, *CLTCL1*, especially stood out compared to the other four genes, having 17 mutations in cases versus 6 mutations in controls (Table 3).

**Table 3:** Whole-exome screen identifies additional potential recessive mutations in the four candidate autism genes.

Gene symbol	SNP	Position	Mutation	Zygosity	Cases	Controls
*UBE3B*	rs61748069	chr12: 108,420,439	S280P	Homozygous	0	1
*UBE3B*	rs61748069	chr12: 108,420,439	S280P	Compound heterozygous	1	0
	var_12_109948232	chr12: 108,432,615	R609C			
*CLTCL1*	rs5748024	chr22: 17,548,288	R1620H	Compound heterozygous	10	4
	rs2073738	chr22: 17,550,956	V1592M			
*CLTCL1*	rs5748024	chr22: 17,548,288	R1620H	Compound heterozygous	1	0
	var_22_19241688	chr22: 17,621,688	A105T			
*CLTCL1*	rs2073738	chr22: 17,550,956	V1592M	Compound heterozygous	1	0
	var_22_19241688	chr22: 17,621,688	A105T			
*CLTCL1*	var_22_19184109	chr22: 17,564,109	R1311Q	Compound heterozygous	1	0
	rs1060374	chr22: 17,593,033	E691K			
*CLTCL1*	var_22_19184113	chr22: 17,564,113	E1310K	Compound heterozygous	1	0
	var_22_19222211	chr22: 17,602,211	E330K			
*CLTCL1*	var_22_19187289	chr22: 17,567,289	V1277I	Compound heterozygous	0	1
	rs117542241	chr22: 17,578,017	N1023I			
*CLTCL1*	rs34486244	chr22: 17,576,615	E1087K	Compound heterozygous	0	1
	rs45489597	chr22: 17,597,422	R574H			
*CLTCL1*	rs35398725	chr22: 17,587,491	K941R	Compound heterozygous	1	0
	rs45489597	chr22: 17,597,422	R574H			
*CLTCL1*	rs5746697	chr22: 17,610,365	K205R	Compound heterozygous	1	0
	var_22_19241688	chr22: 17,621,688	A105T			
*CLTCL1*	var_22_19241688	chr22: 17,621,688	A105T	Compound heterozygous	1	0
	rs3747059	chr22: 17,643,214	P61L			
*NCKAP5L*	var_12_50186544	chr12: 48,472,811	S1189N	Compound heterozygous	1	0
	var_12_50187579	chr12: 48,473,846	A1066S			
*NCKAP5L*	var_12_50187579	chr12: 48,473,846	A1066S	Homozygous	1	0
*NCKAP5L*	var_12_50187579	chr12: 48,473,846	A1066S	Compound heterozygous	0	1
	rs3741554	chr12: 48,476,934	L326M			
*NCKAP5L*	rs3741554	chr12: 48,476,934	L326M	Homozygous	2	0
*ZNF18*	rs117755721	chr17: 11,822,081	S523L	Compound heterozygous	0	1
	rs62621364	chr17: 11,822,223	F476L			
*ZNF18*	rs117755721	chr17: 11,822,081	S523L	Compound heterozygous	0	1
	var_17_11894428	chr17: 11,835,153	P147L			
*ZNF18*	rs62621364	chr17: 11,822,223	F476L	Homozygous	0	1
*ZNF18*	var_17_11881611	chr17: 11,822,336	C438Y	Compound heterozygous	1	0
	var_17_11894428	chr17: 11,835,153	P147L			
*ZNF18*	var_17_11894428	chr17: 11,835,153	P147L	Homozygous	1	0

Summary of the results of sequence analysis of the 4 candidate autism genes in an independent cohort of 418 autism cases and 371 controls from the ARRA Autism Sequencing Consortium. All four genes (*UBE3B*, *CLTCL1*, *NCKAP5L*, and *ZNF18*) were analyzed for recessive mutations, either homozygous or compound heterozygous.

**Figure 3:**
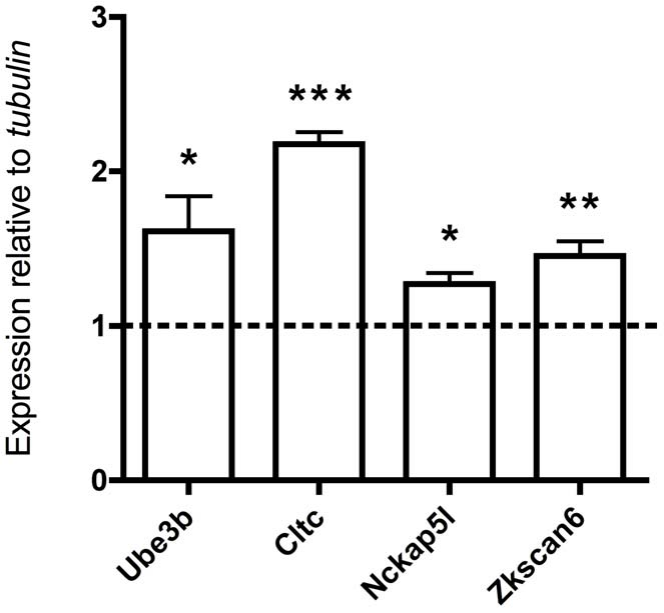
Regulation of four candidate autism genes by neuronal activity. qRT-PCR analysis of total RNA from depolarized mouse cortical neurons stimulated with KCl for 6 hours (the dashed line represents no KCl treatment, values are mean ± SEM from three independent experiments, each experiment was performed in triplicate, ***P<0.0001, **P<0.004, *P<0.04, *t*-test).

Genes with essential roles in synaptic plasticity have been implicated as an important cause of autism (e.g. *NRXN1*, *NLGN3/4X*, *SHANK2/3*) [29], [30], and since many synaptic plasticity genes are regulated by neuronal depolarization [11], [31], we tested the degree to which our autism candidate genes showed expression that could be modulated by neuronal activity. We depolarized mouse cortical neuron cultures and assayed changes in gene expression levels. We found that four out of four of the mouse homologs of our candidate genes are upregulated in response to neuronal activity (*UBE3B*, *CLTCL1*/*Cltc*, *NCKAP5L*, and *ZNF18*/*Zkscan6*) (Figure 3). This is particularly interesting because in general only about 1000 transcripts, or about 3% of the transcriptome, manifest such depolarization-regulated gene transcription [32]. The upregulation of *Ube3b* in response to depolarization resembles the activity-dependent transcription of its close paralog *Ube3a*, which has well-documented roles in synaptic plasticity [26], [27]. The regulation of expression of several potential recessive autism genes by neuronal depolarization suggests that the candidate genes are likely to be involved in neuronal function and/or development, and mutations in these genes might lead to nervous system dysfunction in the context of autism spectrum disorders (ASDs).

In the 12/16 patients for whom we did not identify homozygous candidate mutations, we examined the mutational spectrum under different models of inheritance. Out of an average of 696 rare, heterozygous, and potentially deleterious variants per exome, we identified 67 candidate compound heterozygous changes (at least two deleterious variants in the same gene). Sequenom genotyping validated an average of 27 of these variants, and phasing of the resulting set in trios revealed ∼4 true compound heterozygotes with one allele inherited from each parent. Genotyping of unaffected siblings when available reduced this number to ∼2 variants per individual consistent with fully penetrant, recessive disease (Table S4). For three patients, we narrowed down the candidates to 1 gene and for 8 patients there were no candidate genes with compound heterozygous variants (Table S5). Analysis of X-linked mutations did not identify mutations in well-validated X-linked autism genes, though 11/14 male patients carried rare hemizygous X-linked variants, three of which occurred in genes associated with intellectual disability (*ARHGEF6*, *AFF2*, and *OCRL*). The first variant in *ARHGEF6*, which encodes Rac/Cdc42 guanine nucleotide exchange factor 6, results in an I444N mutation. The second variant in *AFF2*, encoding Fragile X mental retardation 2, causes a P847A mutation that is predicted to be benign by PolyPhen-2. The third variant disrupts a splice donor site in *OCRL* (oculocerebrorenal syndrome of Lowe gene) (Table S6). Splicing mutations in *OCRL* have been identified in patients with Lowe oculocerebrorenal syndrome [33]–[36], characterized by hydrophthalmia, cataract, intellectual disability, vitamin D-resistant rickets, amino aciduria, and reduced ammonia production by the kidney. Since patient AU1019301 is not known to exhibit a renal phenotype or any other Lowe syndrome phenotypes, it is unlikely that this mutation is causative of the neurological condition of the patient. Segregation analysis showed that these three X-linked mutations were inherited from heterozygous mothers, confirming that they are not cell line artifacts. Since our study design enriched for families with potential shared inheritance, it does not permit confident determination of the causative nature of these potential compound heterozygous or X-linked mutations, which could only be tested by analysis of additional cases.

Our results illustrate both the challenges and the potential of whole exome sequencing in an extremely genetically heterogeneous condition such as autism. Each exome contains large numbers of variants that initially challenge analysis. We present a systematic method to approach whole exome data, by filtering for variants compatible with identity by descent, surveying prevalence in controls, segregation analysis, and incorporating functional information (Figure 2). Almost all instances in which new genetic syndromes have been identified using whole exome or whole genome sequencing have involved families with recessive disorders generally (Miller syndrome) [37], [38] and/or shared parental ancestry specifically (*WDR62*-associated cortical malformations) [39], because the analysis of homozygous mutations provides tremendous power to improve “signal to noise” caused by sequencing errors, spontaneous cell line mutations, somatic mutations, etc. Hence, tracing ancestry may be an important tool to define genetic causes in a subset of autism patients. Our study further emphasizes the power of whole exome and whole genome approaches in allowing a complete survey of all potential mutations in the patient genome, and the systematic screening of all major modes of inheritance. Recent studies have confirmed the contribution of *de novo* point mutations (5–20% of cases) [40] and *de novo* copy number variants (5–10% of cases) [41] to autism.

Our data suggest a potentially important role for recessive mutations in autism. Though our pre-selection of 16 patients for whole exome sequencing, and our limited analysis of whole exome data from >400 cases in the ARRA Autism Sequencing Consortium, does not allow us to calculate the proportion of cases likely attributable to recessive as opposed to other causes (e.g. *de novo*, X-linked), our data do suggest that a systematic analysis of recessive causes of autism would be worthwhile. Homozygous null mutations appear to be exceedingly rare in autism, while homozygous missense changes were found in several candidate genes (Table 2), consistent with the possibility that some cases of ASD may reflect hypomorphic mutations in genes that have more severe phenotypes when completely disabled [11]. On the other hand, compound heterozygous recessive mutations could be more common in the outbred families represented by the AGRE.

Furthermore, we find that different patients showed candidate mutations in different ASD candidate genes, confirming that recessive autism genes are likely to be highly heterogeneous. On the other hand, several of the genes we identified represent new neuronal depolarization-dependent genes, further supporting a role of defective synaptic transmission and neuronal plasticity in the pathogenesis of ASD.

Finally, the approach employed here might be of value to the dissection of other complex traits where extreme genetic heterogeneity is suspected or confirmed. Since many neuropsychiatric conditions - including schizophrenia, intellectual disability, and epilepsy - often (albeit not exclusively) arise from loss of gene function, it is reasonable to suppose that recessive loss of gene function may play detectable roles in other conditions. Despite the rich variation in the human exome, our study design and approach to variant prioritization allowed identification of candidate autism genes from a relatively small sample.

## Materials and Methods

### Subjects

Whole exome sequencing was performed on DNA samples from the AGRE collection available at the Broad Institute. All human studies were reviewed and approved by the institutional review board of the Children's Hospital Boston, the Broad Institute, Cambridge, and the local institutions.

### Homozygosity analysis

The analysis was performed using the Illumina 550 SNP genotype data for 1000 families from the AGRE collection. The data was obtained with permission from the AGRE [42]. Runs of homozygosity were calculated using custom scripts, allowing for no more than 2 consecutive heterozygous SNPs in a run and 3 heterozygous calls in every 10 consecutive SNPs. Intervals homozygous for the same haplotype and shared by all affected individuals were used to narrow the locus in each family.

### Estimating relatedness

We used PLINK [23] to calculate the probability that one allele is shared IBD (Z1), and we calculated IBS2*_ratio and the percent of informative SNPs as described by Stevens et al. [24]. Briefly, IBS2*_ratio is equal to (IBS2*)/(IBS2*+IBS0), and the percent of informative SNPs is equal to (IBS0+IBS2*)/(IBS0+IBS1+IBS2), where IBS0 is the total number of observations in which two discordant homozygotes are present, and IBS2* results when two concordant heterozygotes are compared between any pair of individuals.

### Whole-exome sequencing and data analysis

Exome enrichment was performed on 3 µg of genomic DNA, using the SureSelect Human Exome Kit (Agilent Technologies, Inc., Santa Clara, CA), according to the manufacturer's protocol. The kit covers exonic sequences of ∼18,500 genes and a total of ∼33 Mb of target territory. The captured, purified and amplified library targeting the exome from each patient was sequenced on the Illumina GA II. Paired-end sequences were obtained at a read length of 72 bp.

High-throughput sequence analysis was performed according to a customized bioinformatic pipeline for tracking sequence data, aligning reads, calculating coverage, calling variants, annotating variants with respect to functional effect, filtering out benign variation and flagging candidate rare, pathogenic mutations. Briefly, BWA version 0.5.7 (ref. 3) was employed to align reads to the human genome (reference build hg18). Consensus and variant base calls were made with SAMtools version 0.1.7 (pileup), filtered for quality (mapping quality >10 for insertions and deletions, and >25 for SNPs), and loaded into a MySQL database for storage and further processing, including annotation of the predicted consequences (noncoding, coding synonymous, coding nonsynonymous or frameshift, splice site) of each variant using GMCC [43] (Genomic mutation consequence calculator). Candidate mutations were identified by starting with a list of all variants, removing those present either in dbSNP130 or the 1000 Genomes Project database, and selecting for coding nonsynonymous, frameshift or splice site changes. Sequence data were visualized using either the UCSC Genome Browser or the Broad Institute Integrated Genome Viewer. All genomic base positions are presented in reference to the human genome NCBI build 36 (hg18). The functional effect of the mutation on the protein was assessed using PolyPhen-2 [44].

### Sequenom genotyping

Sequenom genotyping of variants in the probands and their family members was performed on the iPLEX Gold platform at the Broad Institute. Variants were genotyped in control individuals also using the Sequenom iPLEX Gold assay at the Molecular Genetics Core Facility at Children's Hospital Boston. The controls collection consisted of 704 neurologically normal samples obtained from the Coriell Cell Repositories (Camden, NJ; 584 Caucasian samples), or available in our lab (80 Saudi and 40 Bedouin samples).

### Resequencing analysis of candidate genes in the ARRA Autism Sequencing Consortium

We screened whole exome sequencing data from a total of 789 exomes (418 autism cases and 371 controls) that were sequenced at the Broad Institute (as described above) as part of a case-control study by the ARRA Autism Sequencing Consortium. Recessive mutations (homozygous and compound heterozygous) were counted in cases and in controls and a Fisher's exact test was used to determine whether the number of mutations in cases was significantly different than the number in controls. Samples in this study are of European ancestry from the AGRE collection, the Autism Sequencing Consortium (ASC), and the National Institute of Mental Health (NIMH).

### Mouse cortical cultures

E16.5 C57B6 mouse embryo cortices were dissected and then dissociated in 1× Hank's Balanced Salt Solution (HBSS), 20 mg/ml trypsin (Worthington Biochemicals, Lakewood, NJ), and 0.32 mg/ml L-cysteine (Sigma, St. Louis, MO) for 10 minutes. Trypsin treatment was terminated with three two-minute washes in 1× HBSS with 10 mg/ml trypsin inhibitor (Sigma, St. Louis, MO). Trituration of cells was performed with a flame-narrowed Pasteur pipette to fully dissociate cells. Neurons were seeded at an approximate density of 1×10^6^/well on 6-well culture plates. The dishes were pre-coated overnight with poly-ornithine (30 µg/mL, Sigma) in water, washed three times with water, and washed once with Neurobasal Medium (Life Technologies, Carlsbad, CA) before use. Neurons were maintained in 2 ml/well Neurobasal Medium containing B27 Supplement (2%; Invitrogen, Carlsbad, CA), penicillin-streptomycin (50 µg/ml penicillin, 50 U/ml streptomycin, Sigma) and glutamine (1 mM, Sigma, St. Louis, MO). Neurons were grown *in vitro* for 7 days. 8 ml of the medium was replaced with 10 ml fresh warm medium on the 4th and 6th days *in vitro* (DIV).

### Membrane depolarization and quantitative RT–PCR detection of activity induction

For KCl depolarization of neurons, DIV 6 neurons were quieted overnight in 1 µM TTX and 100 µM APV, and they were incubated for 0 or 6 hours in 55 mM KCl. Total RNA was isolated from cultures using 1 ml Trizol/well according to the manufacturer's instructions (Invitrogen, Carlsbad, CA). Isolated RNA was treated with DNAseI Amplification Grade (Invitrogen, Carlsbad, CA) and cDNA library was synthesized by cDNA High Capacity cDNA Reverse Transcription Kit (Applied Biosystems, Carlsbad, CA). The cDNA was the source of input for quantitative PCR, using a Step One Plus Real-Time PCR Instrument and SYBR Green reagents (Applied Biosystems, Carlsbad, CA). The relative expression plot was constructed using concentration values that were normalized to corresponding tubulin concentrations.

### Accession numbers

The whole exome sequence data is available online (The National Database for Autism Research (NDAR) Collection ID: NDARCOL0001918).

## Supporting Information

Figure S1 Genetic relatedness. (A) IBS2*_ratio values versus percent of informative SNPs are plotted for all parental pairs with available genotype data from the AGRE collection (red +). Parental pairs from the 16 families where probands were sequenced are indicated (black x). Family identifiers are indicated for some of the 16 families. The majority of these families (AU0708, AU1328, AU0399, AU0222, AU0371, AU0352, AU0005, AU1252, AU1019, AU1196, AU0812) cluster around the average compared to all parental pairs, while some (AU1353, AU0625, AU1388, AU0753) had higher IBS2*_ratio values (particularly AU0753), indicating closer relatedness, and one family had a lower IBS2*_ratio value (AU1261). (B) Relationship of IBS2*_ratio to IBD = 1 (Z1) estimates. Higher Z1 values indicate closer relatedness.
(TIF)

Figure S2 Pedigrees of the 16 AGRE families. Whole exome sequencing was performed on patients indicated with an arrow. Shaded symbols indicate affected individuals.
(TIF)

Figure S3 The rate of validation by Sequenom genotyping correlated with sequencing depth. Pearson's correlation = 0.532, *P* = 0.001×10^−30^, *t*-test.
(TIF)

Table S1 Whole-exome sequencing performance. Average read depth and coverage for each exome are presented. The transition-to-transversion ratios (Ti/Tv) were as expected for coding sequences.
(DOCX)

Table S2 Summary of the variants detected per proband, before and after filtration.
(DOCX)

Table S3 List of genes that were excluded as candidate autism genes. Homozygous variants in these genes were considered benign either because they were not in ROHs, were prevalent in control chromosomes, were not expressed in brain, or the genes are mutated in other disorders. Noncanonical splice site variants were also excluded. Brain expression information is based on data from NIMH Transcriptional Atlas of Human Brain Development.
(DOCX)

Table S4 Summary of compound heterozygous variants per proband, before and after filtration. For each proband, variants that are candidates for being compound heterozygotes were validated. Parental genotypes were used for segregation analysis to determine which variants are true compound heterozygotes.
(DOCX)

Table S5 Candidate autism genes that contain compound heterozygous variants.
(DOCX)

Table S6 Hemizygous variants on the X chromosome.
(DOCX)
