# Supplementary figures and images for: Whole-Exome Sequencing and Homozygosity Analysis Implicate Depolarization-Regulated Neuronal Genes in Autism
Source: PLoS Genet. 2012 Apr 12;8(4):e1002635. doi: 10.1371/journal.pgen.1002635 (PMC3325173; doi:10.1371/journal.pgen.1002635)

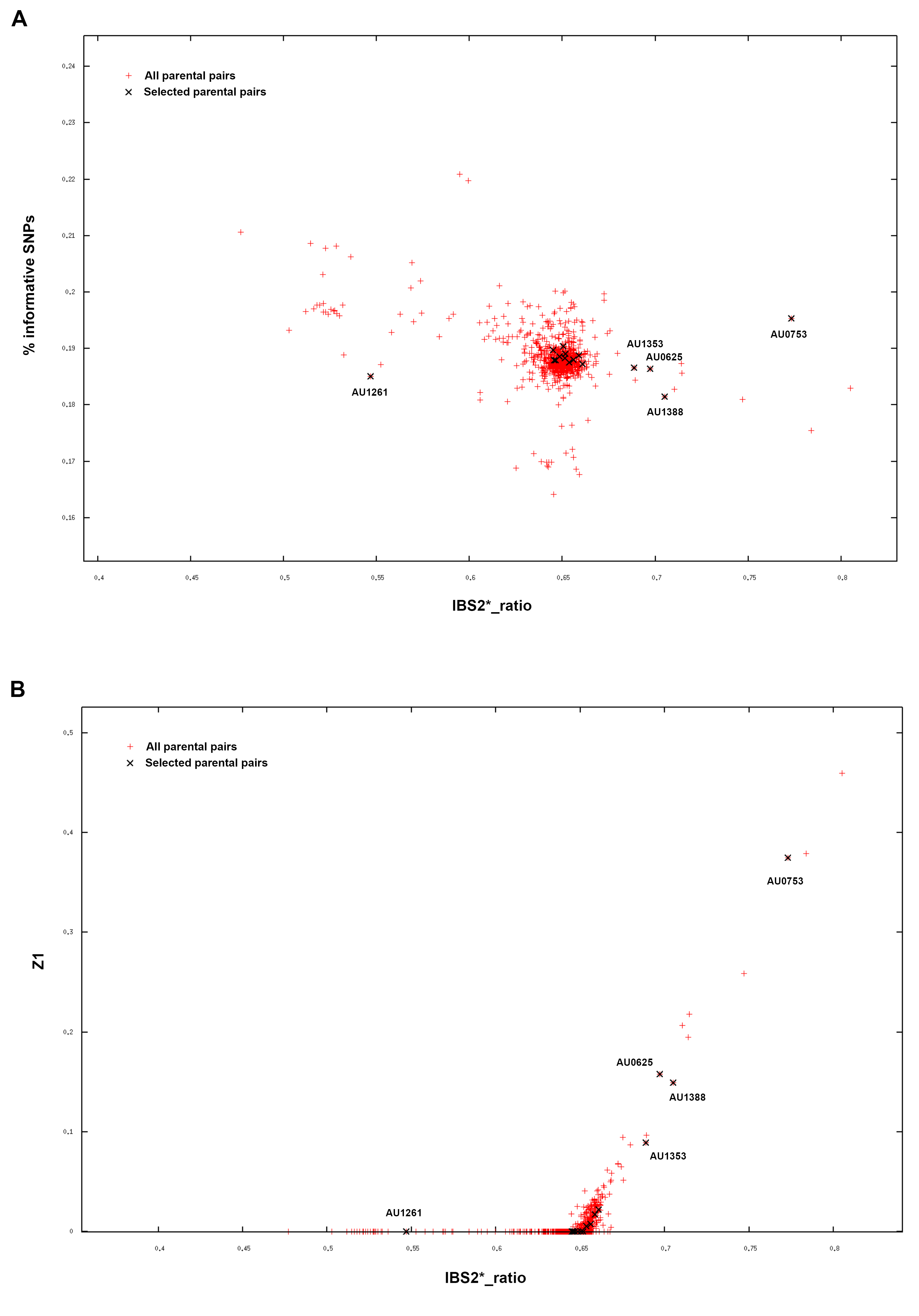

Supplement: Figure S1 — Genetic relatedness. (A) IBS2*_ratio values versus percent of informative SNPs are plotted for all parental pairs with available genotype data from the AGRE collection (red +). Parental pairs from the 16 families where probands were sequenced are indicated (black x). Family identifiers are indicated for some of the 16 families. The majority of these families (AU0708, AU1328, AU0399, AU0222, AU0371, AU0352, AU0005, AU1252, AU1019, AU1196, AU0812) cluster around the average compared to all parental pairs, while some (AU1353, AU0625, AU1388, AU0753) had higher IBS2*_ratio values (particularly AU0753), indicating closer relatedness, and one family had a lower IBS2*_ratio value (AU1261). (B) Relationship of IBS2*_ratio to IBD = 1 (Z1) estimates. Higher Z1 values indicate closer relatedness. (TIF) [file pgen.1002635.s001.tif]

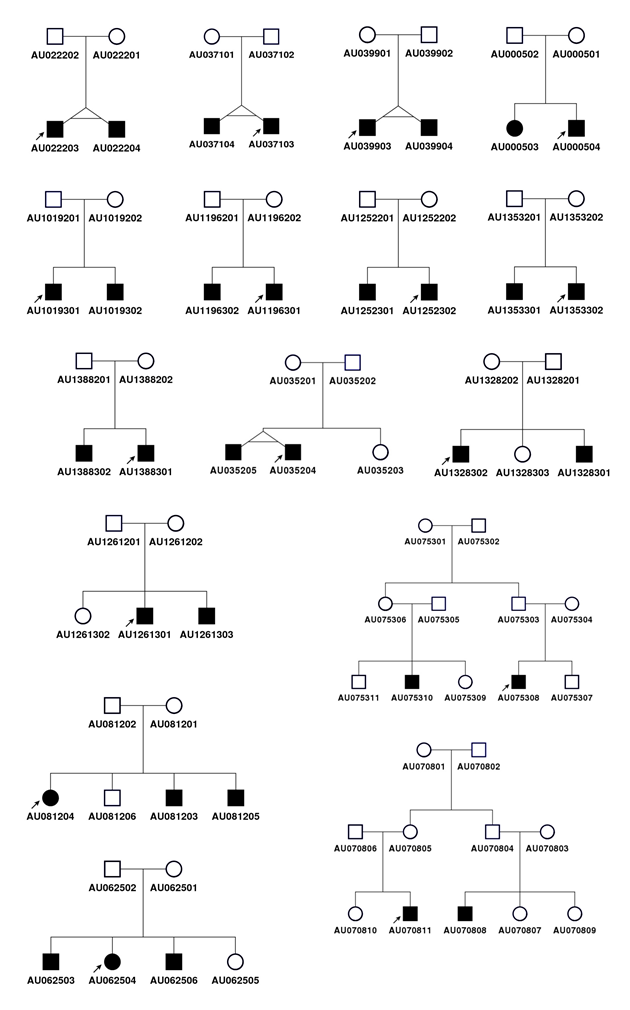

Supplement: Figure S2 — Pedigrees of the 16 AGRE families. Whole exome sequencing was performed on patients indicated with an arrow. Shaded symbols indicate affected individuals. (TIF) [file pgen.1002635.s002.tif]

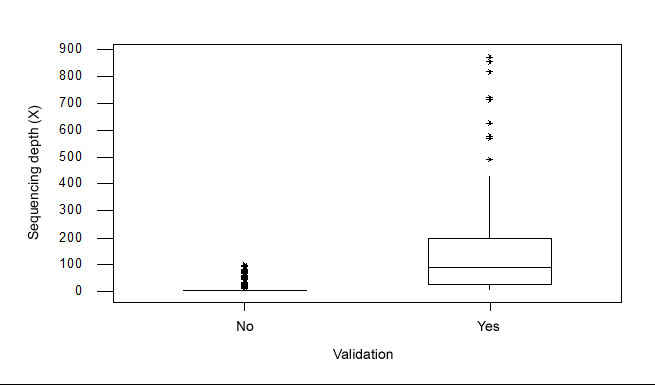

Supplement: Figure S3 — The rate of validation by Sequenom genotyping correlated with sequencing depth. Pearson's correlation = 0.532, P = 0.001×10^−30^, t-test. (TIF) [file pgen.1002635.s003.tif]
