# Supplementary material for: Whole-Exome Sequencing and Homozygosity Analysis Implicate Depolarization-Regulated Neuronal Genes in Autism
Source: PLoS Genet. 2012 Apr 12;8(4):e1002635. doi: 10.1371/journal.pgen.1002635 (PMC3325173; doi:10.1371/journal.pgen.1002635)
Supplement: Table S1 — Whole-exome sequencing performance. Average read depth and coverage for each exome are presented. The transition-to-transversion ratios (Ti/Tv) were as expected for coding sequences. (DOCX) [file pgen.1002635.s004.docx]

**Table S1. Whole exome sequencing performance.**

| **Patient** | **Average read depth (X)** | **Coverage at 20X (%)** | **Ti/Tv** |
| --- | --- | --- | --- |
| AU070811 | 182.87 | 93.10 | 2.80 |
| AU035204 | 217.06 | 91.14 | 2.80 |
| AU081204 | 221.93 | 92.82 | 2.77 |
| AU075308 | 199.96 | 93.13 | 2.68 |
| AU1328302 | 153.99 | 91.92 | 2.76 |
| AU1261301 | 167.20 | 92.72 | 2.78 |
| AU1353302 | 184.21 | 93.10 | 2.75 |
| AU1252302 | 177.01 | 91.56 | 2.76 |
| AU037103 | 197.79 | 93.26 | 2.75 |
| AU1019301 | 192.24 | 91.53 | 2.78 |
| AU1388301 | 206.08 | 91.40 | 2.77 |
| AU1196301 | 210.64 | 92.26 | 2.71 |
| AU022203 | 179.38 | 82.44 | 2.79 |
| AU000504 | 175.94 | 92.47 | 2.76 |
| AU039903 | 177.92 | 91.75 | 2.74 |
| AU062504 | 192.03 | 90.89 | 2.81 |

Average read depth and coverage for each exome are presented. The transition-to-transversion ratios (Ti/Tv) were as expected for coding sequences.
