# Supplementary material for: Whole-Exome Sequencing and Homozygosity Analysis Implicate Depolarization-Regulated Neuronal Genes in Autism
Source: PLoS Genet. 2012 Apr 12;8(4):e1002635. doi: 10.1371/journal.pgen.1002635 (PMC3325173; doi:10.1371/journal.pgen.1002635)
Supplement: Table S2 — Summary of the variants detected per proband, before and after filtration. (DOCX) [file pgen.1002635.s005.docx]

**Table S2. Summary of the variants detected per proband, before and after filtration.**

| **Patient** | **Gender** | **Diagnosis** | **Total variants** | **Novel variants ^a^** | **Novel and potentially pathogenic variants** | **SNPs ^a^** | **Insertions and Deletions ^a^** | **Heterozygous variants ^a^** | **Homozygous variants ^a^** | **Genes ^b^** |
| --- | --- | --- | --- | --- | --- | --- | --- | --- | --- | --- |
| AU070811 | Male | Autism | 35,272 | 2,618 | 828 | 600 (72%) | 228 (28%) | 796 (96%) | 32 (4%) | 29 |
| AU035204 | Male | Autism | 34,418 | 2,587 | 785 | 654 (83%) | 131 (17%) | 737 (94%) | 48 (6%) | 45 |
| AU081204 | Female | Broad Spectrum | 34,103 | 2,454 | 751 | 571 (76%) | 180 (24%) | 722 (96%) | 29 (4%) | 28 |
| AU075308 | Male | Autism | 35,513 | 2,525 | 738 | 612 (83%) | 126 (17%) | 702 (95%) | 36 (5%) | 33 |
| AU1328302 | Male | Autism | 34,944 | 2,415 | 713 | 602 (84%) | 111 (16%) | 675 (95%) | 38 (5%) | 36 |
| AU1261301 | Male | Autism | 34,940 | 2,787 | 856 | 754 (88%) | 102 (12%) | 821 (96%) | 35 (4%) | 34 |
| AU1353302 | Male | Autism | 35,337 | 2,655 | 796 | 666 (84%) | 130 (16%) | 770 (97%) | 26 (3%) | 24 |
| AU1252302 | Male | Autism | 34,579 | 2,412 | 696 | 592 (85%) | 104 (15%) | 664 (95%) | 32 (5%) | 31 |
| AU037103 | Male | Autism | 34,611 | 2,272 | 612 | 508 (83%) | 104 (17%) | 587 (96%) | 25 (4%) | 25 |
| AU1019301 | Male | Autism | 34,471 | 2,498 | 705 | 570 (81%) | 135 (19%) | 652 (92%) | 53 (8%) | 48 |
| AU1388301 | Male | Autism | 34,445 | 2,579 | 723 | 561 (78%) | 162 (22%) | 678 (94%) | 45 (6%) | 44 |
| AU1196301 | Male | Autism | 34,219 | 2,472 | 725 | 581 (80%) | 144 (20%) | 671 (93%) | 54 (7%) | 43 |
| AU022203 | Male | Autism | 32,741 | 2,506 | 744 | 573 (77%) | 171 (23%) | 704 (95%) | 40 (5%) | 36 |
| AU000504 | Male | Autism | 34,510 | 2,279 | 621 | 522 (84%) | 99 (16%) | 580 (93%) | 41 (7%) | 33 |
| AU039903 | Male | Autism | 35,220 | 2,534 | 753 | 639 (85%) | 114 (15%) | 713 (95%) | 40 (5%) | 36 |
| AU062504 | Female | Broad Spectrum | 34,514 | 2,471 | 707 | 597 (84%) | 110 (16%) | 664 (94%) | 43 (6%) | 33 |

^a^ Not in dbSNP130 or the 1000 Genomes project

^b^ Genes with homozygous variants
