# Supplementary material for: Whole-Exome Sequencing and Homozygosity Analysis Implicate Depolarization-Regulated Neuronal Genes in Autism
Source: PLoS Genet. 2012 Apr 12;8(4):e1002635. doi: 10.1371/journal.pgen.1002635 (PMC3325173; doi:10.1371/journal.pgen.1002635)
Supplement: Table S3 — List of genes that were excluded as candidate autism genes. Homozygous variants in these genes were considered benign either because they were not in ROHs, were prevalent in control chromosomes, were not expressed in brain, or the genes are mutated in other disorders. Noncanonical splice site variants were also excluded. Brain expression information is based on data from NIMH Transcriptional Atlas of Human Brain Development. (DOCX) [file pgen.1002635.s006.docx]

**Table S3.** **List of genes that were excluded as candidate autism genes.**

| **Patient** | **Gene symbol** | **Gene name** | **Mutation** | **Effect** | **Prevalence in control chromosomes** | **PolyPhen-2 prediction (score)** | **Exclusion criteria** |
| --- | --- | --- | --- | --- | --- | --- | --- |
| AU070811 | *ESPN* | Espin | chr1: 6,434,661 G>A | R182Q | N/A | Probably damaging (0.997) | Mutated in hearing loss ^b^ |
| AU070811 | *PSG7* | Pregnancy specific beta-1-glycoprotein 7 | chr19: 48,125,516 G>C | N208K | 109/1436 (7.6%) (0 homozygotes) | Benign (0.000) | Not in ROHs |
| AU035204 | *BPIL2* | Bactericidal/permeability-increasing protein-like 2 | chr22: 31,173,317 T>C | S86G | N/A | Benign (0.005) | Not in ROHs |
| AU035204 | *CRY1* | Cryptochrome 1 (photolyase-like) | chr12: 105,910,870 T>G | Splice site | 28/1338 (2.0%) (11 homozygotes) | N/A | High prevalence in controls |
| AU035204 | *PLA2G12A* | Phospholipase A2, group XIIA | chr4: 110,858,149 T>A | Splice site | 8/1326 (0.6%) (0 homozygotes) | N/A | Noncanonical splice site |
| AU035204 | *SART3* | Squamous cell carcinoma antigen recognized by T cells 3 | chr12: 107,462,359 C>T | R252Q | 1/1344 (0.07%) (0 homozygotes) | Probably damaging (0.989) | Mutated in disseminated superficial actinic porokeratosis ^c^ |
| AU075308 | *TTC30A* | Tetratricopeptide repeat domain 30A | chr2: 178,190,840 G>T | P279H | 320/1482 (23.1%) (40 homozygotes) | Probably damaging (0.999) | Not in ROHs; high prevalence in controls |
| AU075308 | *WDR85* | WD repeat domain 85 | chr9: 139,569,822 C>T | R174Q | 54/1390 (4.9%) (30 homozygotes) | Benign (0.001) | High prevalence in controls |
| AU1353302 | *PIK3R6* | Phosphoinositide-3-kinase, regulatory subunit 6 | chr17: 8,672,874 C>A | A350S | 24/1382 (1.7%) (1 homozygote) | Benign (0.000) | Not in ROHs |
| AU1252302 | *GSDMC* | Gasdermin C | chr8: 130,832,965 C>T | Splice site | 32/760 (4.2%) (1 homozygote) | N/A | Not in ROHs; no brain expression |
| AU037103 | *GEMIN4* | Gem (nuclear organelle) associated protein 4 | chr17: 595,414 G>T | H873Q | 42/1260 (3.0%) (0 homozygotes) | Benign (0.033) | High prevalence in controls |
| AU1019301 | *C12orf35* | Hypothetical protein LOC55196 | chr12: 32,027,291 A>G | H712R | 0/764 (0.0%) | Probably damaging (0.983) | Not in ROHs |
| AU1019301 | *RASA2* | RAS p21 protein activator 2 | chr3: 142,781,912 A>G | I535V | 2/1334 (0.14%) (0 homozygotes) | Benign (0.000) | Very low brain expression |
| AU022203 | *BGN* | Biglycan | chrX: 152,423,908 G>A | K86R | 71/1348 (5.3%) (26 homo/hemizygotes) | Benign (0.000) | Not in ROHs; high prevalence in controls |
| AU039903 | *GEM* | GTP binding protein overexpressed in skeletal muscle | chr8: 95,341,725 G>C | D61E | 0/1388 (0.0%) | Benign (0.001) | Very low brain expression |

Homozygous variants in these genes were considered benign either because they were not in ROHs, were prevalent in control chromosomes, were not expressed in brain, or the genes are mutated in other disorders. Noncanonical splice site variants were also excluded. Brain expression information is based on data from NIMH Transcriptional Atlas of Human Brain Development ^a^.

ROHs: Runs of homozygosity

^a^ NIMH Transcriptional Atlas of Human Brain Development [Internet]. Funded by ARRA Awards 1RC2MH089921-01, 1RC2MH090047-01, and 1RC2MH089929-01. Available from: http://developinghumanbrain.org.

^b^ Naz et al., 2004; Donaudy et al., 2006

^c^ Zhang et al., 2005
