# Supplementary material for: Whole-Exome Sequencing and Homozygosity Analysis Implicate Depolarization-Regulated Neuronal Genes in Autism
Source: PLoS Genet. 2012 Apr 12;8(4):e1002635. doi: 10.1371/journal.pgen.1002635 (PMC3325173; doi:10.1371/journal.pgen.1002635)
Supplement: Table S4 — Summary of compound heterozygous variants per proband, before and after filtration. For each proband, variants that are candidates for being compound heterozygotes were validated. Parental genotypes were used for segregation analysis to determine which variants are true compound heterozygotes. (DOCX) [file pgen.1002635.s007.docx]

**Table S4. Summary of compound heterozygous variants per proband, before and after filtration.**

| **Patient** | **Compound heterozygous variants** | **Compound heterozygous variants *** | **Compound heterozygous variants **** | **Validated ^a^ compound heterozygous variants** | **Validated ^a^ compound heterozygous variants that are true compound heterozygotes** | **Validated ^a^ true compound heterozygous variants that segregate with disease** | **Corresponding number of genes** |
| --- | --- | --- | --- | --- | --- | --- | --- |
| AU070811 | 72 | 44 (61%) | 35 (80%) | 23 (66%) | 4 | 4 | 2 |
| AU035204 | 68 | 45 (66%) | 37 (82%) | 26 (70%) | 4 | 4 | 2 |
| AU081204 | 68 | 48 (71%) | 46 (96%) | 32 (70%) | 0 | 0 | 0 |
| AU075308 | 54 | 37 (69%) | 32 (86%) | 20 (62%) | 8 | 6 | 3 |
| AU1328302 | 72 | 49 (68%) | 43 (88%) | 31 (72%) | 2 | 0 | 0 |
| AU1261301 | 82 | 45 (55%) | 39 (87%) | 27 (69%) | 6 | 0 | 0 |
| AU1353302 | 80 | 58 (72%) | 55 (95%) | 39 (71%) | 4 | 2 | 1 |
| AU1252302 | 56 | 28 (50%) | 23 (82%) | 19 (83%) | 2 | 0 | 0 |
| AU037103 | 40 | 26 (65%) | 25 (96%) | 14 (56%) | 2 | 2 | 1 |
| AU1019301 | 58 | 29 (50%) | 25 (86%) | 20 (80%) | 6 | 4 | 2 |
| AU1388301 | 70 | 50 (71%) | 43 (86%) | 37 (86%) | 2 | 0 | 0 |
| AU1196301 | 74 | 56 (76%) | 52 (93%) | 41 (79%) | 4 | 0 | 0 |
| AU022203 | 90 | 67 (74%) | 63 (94%) | 32 (51%) | 2 | 2 | 1 |
| AU000504 | 54 | 36 (67%) | 33 (92%) | 21 (64%) | 8 | 0 | 0 |
| AU039903 | 66 | 49 (74%) | 42 (86%) | 20 (48%) | 6 | 6 | 3 |
| AU062504 | 68 | 41 (60%) | 33 (80%) | 23 (70%) | 1 | 0 | 0 |

For each proband, variants that are candidates for being compound heterozygotes were validated. Parental genotypes were used for segregation analysis to determine which variants are true compound heterozygotes.

* Successful Sequenom design

** Successful Sequenom run

^a^ Variants validated by Sequenom analysis
