# Supplementary material for: Whole-Exome Sequencing and Homozygosity Analysis Implicate Depolarization-Regulated Neuronal Genes in Autism
Source: PLoS Genet. 2012 Apr 12;8(4):e1002635. doi: 10.1371/journal.pgen.1002635 (PMC3325173; doi:10.1371/journal.pgen.1002635)
Supplement: Table S5 — Candidate autism genes that contain compound heterozygous variants. (DOCX) [file pgen.1002635.s008.docx]

**Table S5. Candidate autism genes that contain compound heterozygous variants.**

| **Patient** | **Gene symbol** | **Gene name** | **Mutations** | | **Effect** | **PolyPhen-2 prediction (score)** |
| --- | --- | --- | --- | --- | --- | --- |
| AU070811 | *CCBP2* | Chemokine binding protein 2 | chr3: 42,881,599 G>A | chr3: 42,881,651 G>C | G201R / G218A | Probably damaging (0.985) / Probably damaging (0.999) |
| AU070811 | *C2orf43* | Hypothetical protein LOC60526 | chr2: 20,838,170 C>T | chr2: 20,853,553 T>C | E117K / S81G | Possibly damaging (0.703) / Possibly damaging (0.672) |
| AU035204 | *TRIP12* | Thyroid hormone receptor interactor 12 | chr2: 230,409,913 C>T | chr2: 230,433,438 T>C | A44T / K51R | Benign (0.138) / Benign (0.020) |
| AU035204 | *LSG1* | Large subunit GTPase 1 homolog (S. cerevisiae) | chr3: 195,846,649 C>T | chr3: 195,868,274 delTTC | G580D / E126V | Benign (0.025) / Possibly damaging (0.408) |
| AU081204 | N/A | N/A | N/A | N/A | N/A | N/A |
| AU075308 | *SMG6* | Smg-6 homolog, nonsense mediated mRNA decay factor (C. elegans) | chr17: 2,149,230 T>C | chr17: 2,150,277 G>T | T492A / Q143K | Benign (0.000) / Possibly damaging (0.403) |
| AU075308 | *EP400* | E1A binding protein p400 | chr12: 131,076,233 G>A | chr12: 131,088,484 C>T | A1649T / P2033S | Unknown (not available) |
| AU075308 | *CRTAC1* | Cartilage acidic protein 1 | chr10: 99,645,649 C>T | chr10: 99,761,020 C>T | V224I / R30Q | Benign (0.024) / Benign (0.058) |
| AU1328302 | N/A | N/A | N/A | N/A | N/A | N/A |
| AU1261301 | N/A | N/A | N/A | N/A | N/A | N/A |
| AU1353302 | *ITIH3* | Inter-alpha (globulin) inhibitor H3 | chr3: 52,809,648 G>A | chr3: 52,811,733 A>G | R377Q / D527G | Benign (0.135) / Benign (0.003) |
| AU1252302 | N/A | N/A | N/A | N/A | N/A | N/A |
| AU037103 | *ITGA1* | Integrin, alpha 1 | chr5: 52,225,320 A>C | chr5: 52,276,607 C>A | K328Q / S1121R | Possibly damaging (0.232) / Benign (0.109) |
| AU1019301 | *B4GALNT3* | Beta-1,4-N-acetyl-galactosaminyl transferase 3 | chr12: 533,139 G>A | chr12: 538,809 G>A | A146T / R499Q | Benign (0.000) / Possibly damaging (0.645) |
| AU1019301 | *NPBWR2* | Neuropeptides B/W receptor 2 | chr20: 62,207,827 C>T | chr20: 62,208,411 G>A | V268M / A73V | Probably damaging (0.997) / Benign (0.054) |
| AU1388301 | N/A | N/A | N/A | N/A | N/A | N/A |
| AU1196301 | N/A | N/A | N/A | N/A | N/A | N/A |
| AU022203 | *MTTP* | Microsomal triglyceride transfer protein | chr4: 100,751,625 G>A | chr4: 100,761,331 G>C | G661S / L811F | Benign (0.000) / Possibly damaging (0.842) |
| AU000504 | N/A | N/A | N/A | N/A | N/A | N/A |
| AU039903 | *LOC89944 / GLB1L2* | Galactosidase, beta 1-like 2 | chr11: 133,717,976 C>A | chr11: 133,731,411 C>T | H69N / R189C | Probably damaging (0.997) / Probably damaging (0.976) |
| AU039903 | *STAB2* | Stabilin 2 | chr12: 102,570,485 A>G | chr12: 102,671,214 G>A | N427D / E2223K | Benign (0.092) / Probably damaging (0.968) |
| AU039903 | *HHATL* | Hedgehog acyltransferase-like | chr3: 42,710,283 A>G | chr3: 42,714,733 G>A | S360P / R200C | Possibly damaging (0.214) / Probably damaging (0.997) |
| AU062504 | N/A | N/A | N/A | N/A | N/A | N/A |
