# Supplementary material for: Whole-Exome Sequencing and Homozygosity Analysis Implicate Depolarization-Regulated Neuronal Genes in Autism
Source: PLoS Genet. 2012 Apr 12;8(4):e1002635. doi: 10.1371/journal.pgen.1002635 (PMC3325173; doi:10.1371/journal.pgen.1002635)
Supplement: Table S6 — Hemizygous variants on the X chromosome. (DOCX) [file pgen.1002635.s009.docx]

**Table S6.** **Hemizygous variants on the X chromosome.**

| **Patient** | **Gender** | **Validated ^a^ X-linked variants that segregate with disease** | **Gene symbol** | **Gene name** | **Mutation** | **Effect** | **PolyPhen-2 prediction (score)** |
| --- | --- | --- | --- | --- | --- | --- | --- |
| AU070811 | Male | 5 | *CXCR3* | Chemokine (C-X-C motif) receptor 3 | chrX: 70,753,057 C>A | W330C | Possibly damaging (0.840) |
|  |  |  | *NOX1* | NADPH oxidase 1 isoform long variant | chrX: 100,005,218 G>A | A55V | Probably damaging (0.997) |
|  |  |  | *ENOX2* | Ecto-NOX disulfide-thiol exchanger 2 isoform a | chrX: 129,593,189 C>T | E488K | Possibly damaging (0.363) |
|  |  |  | *MAGEC3* | Melanoma antigen family C, 3 isoform 2 | chrX: 140,812,984 C>G | L294V | Probably damaging (0.982) |
|  |  |  | *GAB3* | GRB2-associated binding protein 3 | chrX: 153,593,889 G>A | S292F | Possibly damaging (0.391) |
| AU035204 | Male | 6 | *GLRA2* | Glycine receptor, alpha 2 | chrX: 14,658,430 A>G | I421V | Benign (0.000) |
|  |  |  | *ZFX* | Zinc finger protein, X-linked | chrX: 24,107,409 G>C | E83Q | Possibly damaging (0.629) |
|  |  |  | *CCNB3* | Cyclin B3 isoform 3 | chrX: 50,069,477 C>T | P523L | Possibly damaging (0.602) |
|  |  |  | *BEX2* | Brain expressed X-linked 2 | chrX: 102,451,367 T>C | Y65C | Benign (0.021) |
|  |  |  | *SERPINA7* | Serine (or cysteine) proteinase inhibitor, clade A, member 7 | chrX: 105,164,151 G>A | A415V | Benign (0.000) |
|  |  |  | *ARHGEF6 ^b^* | Rac/Cdc42 guanine nucleotide exchange factor 6 | chrX: 135,595,563 A>T | I444N | Probably damaging (0.984) |
| AU081204 | Female | 0 | N/A | N/A | N/A | N/A | N/A |
| AU075308 | Male | 1 | *ARMCX1* | Armadillo repeat containing, X-linked 1 | chrX: 100,694,786 G>T | D73Y | Possibly damaging (0.364) |
| AU1328302 | Male | 3 | *MAGIX* | PDZ domain containing, X chromosome | chrX: 48,908,254 G>A | R71H | Probably damaging (0.991) |
|  |  |  | *BMP15* | Bone morphogenetic protein 15 | chrX: 50,670,725 C>T | R68W | Probably damaging (1.000) |
|  |  |  | *AFF2 ^b^* | Fragile X mental retardation 2 | chrX: 147,847,654 C>G | P847A | Benign (0.002) |
| AU1261301 | Male | 2 | *DCX* | Doublecortin isoform c | chrX: 110,540,125 T>C | K53R | Benign (0.120) |
|  |  |  | *SLC6A14* | Solute carrier family 6 (amino acid transporter), member 14 | chrX: 115,498,333 T>C | I428T | Benign (0.058) |
| AU1353302 | Male | 0 | N/A | N/A | N/A | N/A | N/A |
| AU1252302 | Male | 1 | *PHKA2* | Phosphorylase kinase, alpha 2 (liver) | chrX: 18,854,555 C>T | A466T | Benign (0.014) |
| AU037103 | Male | 5 | *CTPS2* | Cytidine triphosphate synthase II | chrX: 16,626,996 C>T | R103H | Benign (0.004) |
|  |  |  | *ZNF182* | Zinc finger protein 21 isoform 1 | chrX: 47,727,300 G>T | N94K | Benign (0.004) |
|  |  |  | *HEPH* | Hephaestin isoform a | chrX: 65,339,961 G>A | S703N | Benign (0.000) |
|  |  |  | *STARD8* | START domain containing 8 | chrX: 67,854,095 G>A | R125H | Benign (0.001) |
|  |  |  | *OR13H1* | Olfactory receptor, family 13, subfamily H, member 1 | chrX: 130,505,748 C>A | T7K | Possibly damaging (0.833) |
| AU1019301 | Male | 1 | *OCRL ^b^* | Phosphatidylinositol polyphosphate 5-phosphatase | chrX: 128,536,911 G>A | Splice site | N/A |
| AU1388301 | Male | 1 | *ATP6AP1* | ATPase, H+ transporting, lysosomal accessory protein 1 | chrX: 153,310,652 A>G | T76A | Possibly damaging (0.792) |
| AU1196301 | Male | 0 | N/A | N/A | N/A | N/A | N/A |
| AU022203 | Male | 3 | *SHROOM2* | Apical protein of Xenopus-like | chrX: 9,865,467 G>A | R1294H | Possibly damaging (0.807) |
|  |  |  | *MED14* | Mediator complex subunit 14 | chrX: 40,408,598 G>T | P20H | Probably damaging (0.909) |
|  |  |  | *PNMA3* | Paraneoplastic neuronal antigen MA3 | chrX: 151,976,933 G>A | V289M | Probably damaging (0.948) |
| AU000504 | Male | 0 | N/A | N/A | N/A | N/A | N/A |
| AU039903 | Male | 6 | *ARSF* | Arylsulfatase F | chrX: 3,012,678 G>T | M267I | Benign (0.047) |
|  |  |  | *CXorf22* | Hypothetical protein LOC170063 | chrX: 35,917,385 A>T | E914D | Benign (0.029) |
|  |  |  | *SSX7* | Synovial sarcoma, X breakpoint 7 | chrX: 52,696,154 A>G | C102R | Benign (0.000) |
|  |  |  | *TAF7L* | TATA box binding protein-associated factor, RNA polymerase II, Q | chrX: 100,434,509 C>T | E61K | Benign (0.000) |
|  |  |  | *SUHW3* | Suppressor of hairy wing homolog 3 | chrX: 129,205,321 C>A | R93L | Possibly damaging (0.456) |
|  |  |  | *IDH3G* | Isocitrate dehydrogenase 3 (NAD+) gamma | chrX: 152,704,535 C>T | A332T | Probably damaging (0.907) |
| AU062504 | Female | 0 | N/A | N/A | N/A | N/A | N/A |

* Successful Sequenom design

** Successful Sequenom run

^a^ Variants validated by Sequenom analysis

^b^ Genes associated with mental retardation
